# A Comparative Study of the Chemical Composition by SPME-GC/MS and Antiradical Activity of Less Common *Citrus* Species

**DOI:** 10.3390/molecules26175378

**Published:** 2021-09-04

**Authors:** Sara Vitalini, Marcello Iriti, Vittorio Vinciguerra, Stefania Garzoli

**Affiliations:** 1 Department of Agricultural and Environmental Sciences, Università degli Studi di Milano, 20133 Milan, Italy; sara.vitalini@unimi.it; 2 Phytochem Lab, Department of Agricultural and Environmental Sciences, Università degli Studi di Milano, 20133 Milan, Italy; 3 National Interuniversity Consortium of Materials Science and Technology (INSTM), 50121 Firenze, Italy; 4 Center for Studies on Bioispired Agro-Environmental Technology (BAT Center), Università degli Studi di Napoli ‘Federico II’, 80055 Portici, Italy; 5 Department for Innovation in Biological Systems, Food and Forestry, University of Tuscia, 01100 Viterbo, Italy; vittorio.vinciguerra@unitus.it; 6 Department of Drug Chemistry and Technology, Sapienza University, 00185 Rome, Italy

**Keywords:** *Citrus*, SPME, volatile compounds, polyphenols, flavonoids, ABTS, DPPH, PCA

## Abstract

Citrus secondary metabolites, such as terpene compounds, are very important for human health due to their bioactivity including anti-inflammatory, anti-cancer, and antioxidant effects. In this work, for the first time, the volatile chemical composition of peels and juices from four different *Citrus* species (*C. junos*, *Citrus × aurantium*, *C. aurantium* ‘Bizzarria’ and *C. medica* ‘Florentina’, commonly known as Yuzu jeune, Oni Yuzu, Bizzarria orange and Florence cedar, respectively) was investigated by Solid-Phase Microextraction-Gas Chromatography/Mass Spectrometry (SPME-GC/MS) technique and the antiradical activity was also examined. The results showed that limonene and γ-terpinene were the main volatile substances detected both in the juices and in the peels, followed by other minority compounds responsible for the phyto-complex of the unique aromas which characterize each individual analyzed *Citrus* species. Principal component analysis (PCA), performed on volatile compounds, showed both some correlation as well as a clear separation between the juice and the peel of each species. Among them, Oni Yuzu juice was found to be the richest in total polyphenols and flavonoids while its capacity to scavenge ABTS^•+^ and DPPH^•^ radicals was similar to that of Yuzu Jeune and Bizzarria orange.

## 1. Introduction

Fruits are one of the main food products of the world and citrus fruits certainly represent the largest portion [1]. Their main use is in the food and culinary sector, but they are also widely used in the medicinal and cosmetic fields thanks to their countless beneficial properties such as antioxidant and vasoprotective effects. Citrus fruits also act as stimulants of microcirculation and the immune system [2,3].

*Citrus* is a genus of flowering plants belonging to the Rutaceae family native to Southeast Asia and widely distributed in the world, and are among the oldest fruit crops to be domesticated [4]. In addition to the most common and well-known citrus fruits, there are some unusual ones that have begun to garner worldwide attention, both in the culinary and perfumery fields. Among these, *Citrus junos* Siebold ex Tanaka (Yuzu Jeune), which originated in China as a natural cross between *C. reticulata* Blanco and *C. cavaleriei* H. Lév. ex Cavalerie, has a thick and lumpy peel, is yellow when ripe with little pulp, and has numerous large seeds inside. Its taste is slightly sour, intermediate between grapefruit and mandarin. This citrus fruit, oblate to nearly pyriform and 4–8 cm in diameter, is widely used in Japanese cuisine, but rarely consumed as a fresh fruit [5]. *Citrus × aurantium* L. (syn., *C*. × *pseudogulgul* Shirai) (Oni Yuzu) is also from China, only later introduced to Japan, but supposedly unrelated to the *Yuzu Jeune* and probably a cross between *C. medica* and *C. hystrix* DC. The fruit resembles a large (around 20 cm in diameter) lumpy yellow citron with a thick and wrinkled peel but of good flavor and rich in essential oils. It may contain very few or no seeds, and the acidic pulp is dry rather than juicy. *Oni Yuzu* is suitable for marmalade and jam, but not as raw food [5]. From Italy, *C. aurantium* ‘Bizzarria’ (Bizzarria orange) is a variety with Tuscan origins, part of the Medici collections, described for the first time in 1664 and rediscovered near Florence in the early 1900s. It is a periclinal chimera, a rare and very particular hybrid obtained from the cross between *C. × aurantium* L. and *C. limon* (L.) Osbeck. The fruits with intermediate morphological characteristics of both species are lumpy with longitudinal bands of different colors, from green to yellow or orange. It is used as a classic bitter orange or a lemon [6,7]. Lastly, *C. medica* ‘Florentina’ (Florence cedar) is another variety of the Medici collections discovered at the beginning of the 17th century in Florence. It is a very fragrant fruit, good to eat, and of medium size with an elongated and pointed apex. The pulp is juicy, acidic and delicate while the peel is golden-yellow in color and thick and wrinkled [8].

The uses of these *Citrus* species are various, especially in the food sector. In particular, the peels are used to intensify the flavors of the fruit or for infusion by inserting them into tea bags; particular cuts of the peels are perfect for industries such as food services and the baked confectionery sector [9]. The juices are widely used in the catering sector to make 100% natural products. One of the most recent applications concerns the use of dehydrated citrus, thus ensuring the conservation of all their beneficial properties. Due to the various real and potential uses of these citrus fruits, it would certainly be interesting and useful to know their volatile composition.

In general, citrus fruits have a very unique and intense aroma, which makes them particularly pleasing to the nose. The flavor is determined mainly by the chemical senses of taste and smell, and only a limited number of volatile components contribute to aroma characteristics. Generally, the compounds present in small concentrations are more important in determining the flavor compared to those in high concentration [10]. Furthermore, the quantities of these compounds are sometimes so low as to be difficult to detect, which is why, thanks to a pre-concentration phase, the headspace sampling technique is the most suitable for a more accurate investigation of the aromatic profile of one food or another.

**Figure 1:**
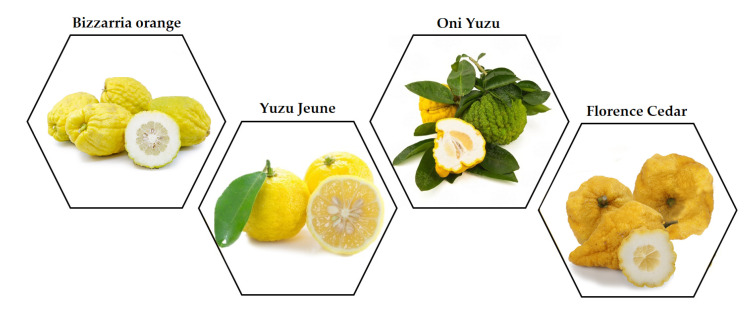
Images of *Citrus* species investigated.

In this work, for the first time, the chemical analysis of peels and juices of four uncommon *Citrus* species (Yuzu Jeune, Oni Yuzu, Bizzarria orange and Florence cedar) (Figure 1), grown in Switzerland, was performed by SPME-GC/MS technique with the aim of characterizing and comparing, both from a qualitative and quantitative point of view, their volatile composition. This solvent-free extraction method is relatively simple and does not require any kind of sample pre-treatment, thus also avoiding any loss of volatile compounds and reflecting the true native volatile profile of the citrus fruits without any alteration [11,12]. The antiradical properties of citrus juices were also investigated by the ABTS^•+^ and DPPH^•^ assays after determining the content of total phenolics and flavonoids.

## 2. Results

### 2.1. Juice and Peel Vapor Phase Chemical Volatile Composition

SPME-GC/MS technique was used to investigate the vapor phase chemical composition of Yuzu Jeune, Oni Yuzu, Bizzarria orange and Florence cedar juices and peels. The relative percentages of the identified compounds in are reported in the Table 1. Monoterpene hydrocarbons (99.08% and 96.58%) were the principal components followed by a small percentage of oxygenated monoterpenes (0.76% and 2.81%) and sesquiterpene hydrocarbons (0.16% and 0.53%) in the vapor phase of Yuzu Jeune juice and peel, respectively. Limonene was the most abundant monoterpene detected both in the juice (78.25%) and in the peel (78.40%) followed by γ-terpinene (14.43% and 11.43%). Additionally, α-Thujene (0.69%; 0.68%) and *p*-cymene (2.38%; 2.33%) were revealed in similar percentage values in the juice and peel, respectively. *Cis*-β-ocimene (0.42%), linalool, formate (2.81%) and *cis*-β-farnesene (0.53%) were detected only in the peel. On the contrary, terpinolene (0.46%), 4-terpinenyl acetate (0.76%) and *cis*-α-bergamotene (0.16%) were present only in the juice.

The vapor phase chemical composition of Oni Yuzu juice and peel was characterized by monoterpene hydrocarbons (99.84% and 99.68%, respectively), while the oxygenated monoterpenes were missing. In addition, in Oni Yuzu limonene reached the highest percentage values both in the juice (85.74%) and in the peel (84.15%) followed by γ-terpinene (9.46% and 9.18%). Furthermore, α-Thujene (0.20%), α-phellandrene (0.19%) and β-ocimene (0.58%) were detected only in the juice while α-pinene (0.80%), α-terpinene (0.15%) and α-farnesene (0.10%), a sesquiterpene hydrocarbon, were only in the peel.

Twenty-five molecules were identified in Bizzarria orange juice and peel. Monoterpene hydrocarbons (98.67%; 95.12%), oxygenated monoterpenes (0.79%; 3.64%) and sesquiterpenes (0.38%; 0.68%) were found in both the juice and the peel. Limonene (70.85%; 75.40%) and γ-terpinene (21.51%; 13.36%) were the most abundant components of the juice and peel vapor phase, respectively. Traces of some compounds such as β-citronellol, perillal, α-copaene, germacrene D and δ-cadinene were detected in peels.

Additionally, Florence cedar juice and peel were characterized by limonene (55.16%; 54.80%) and γ-terpinene (35.21%; 26.89%) as principal components among monoterpene hydrocarbons. Other minor compounds such as α-pinene (1.25%; 3.15%) and *p*-cymene (1.23%; 2.34%) were identified in the juice and peel. The oxygenated monoterpenes and the sesquiterpenes reached 5.61% and 1.28% in the peel and 1.4% and 0.77% in the juice, respectively.

Summing up, limonene, α-terpinene, *p*-cymene and α-pinene were the major compounds detected in all juices and peels of the investigated *Citrus* species. On the other hand, the volatile chemical compositions of the juice and the peel differed from each other in the presence or absence and in a different trend of minority compounds which contribute enormously totheir characteristic aroma and flavor.

**Figure 2:**
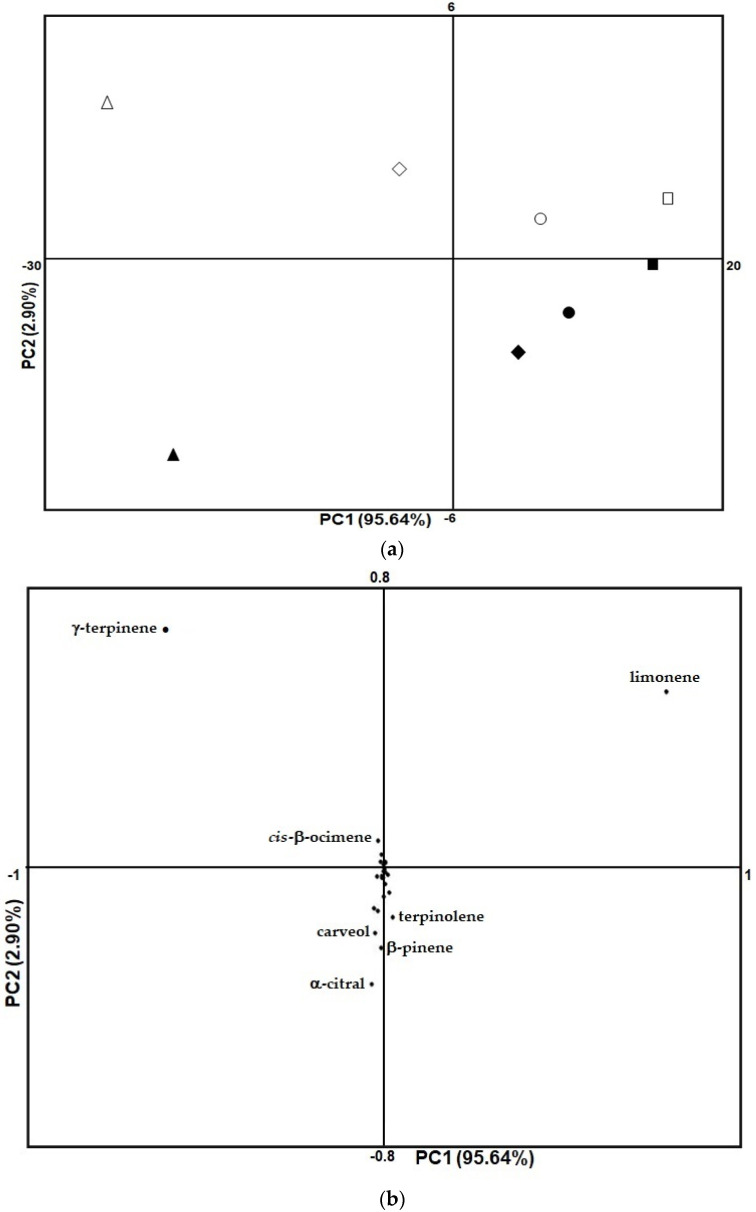
Score plot of citrus juice (outlined symbols) and peel (filled symbols) samples. Circle: Yuzu Jeune, square: Oni Yuzu, triangle: Florence cedar, diamond: Bizzarria orange. (**a**). Loading plot of volatile products extracted from citrus samples (juice and peel) (**b**).

All the relative percentages of the identified compounds listed in Table 1 were used to carry out a principal component analysis (PCA) to display differences and similarities between citrus samples. The first two extracted principal components, PC1 and PC2 explained, respectively, 95.64% and 2.90% of variance for a total of 98.54% as clearly shown in the score plot (Figure 2a). In fact, the four species under examination differ almost exclusively along the PC1; in particular, the Florence cedar appears to be the least similar to the other three in regards the composition of volatile compounds.

The differentiation between the four species showed the same trend considering both the volatile compounds of the juices and those of the peels. In addition, there is a clear separation between the juice samples all arranged between the first and second quadrants, and the peel samples, arranged in opposite quadrants (third and fourth) (Figure 2a). In this regard, the differentiation between juice and peel is much wider in the Florence cedar and to a slightly lesser extent in the Bizzarria orange than in the other two species.

**Figure 3:**
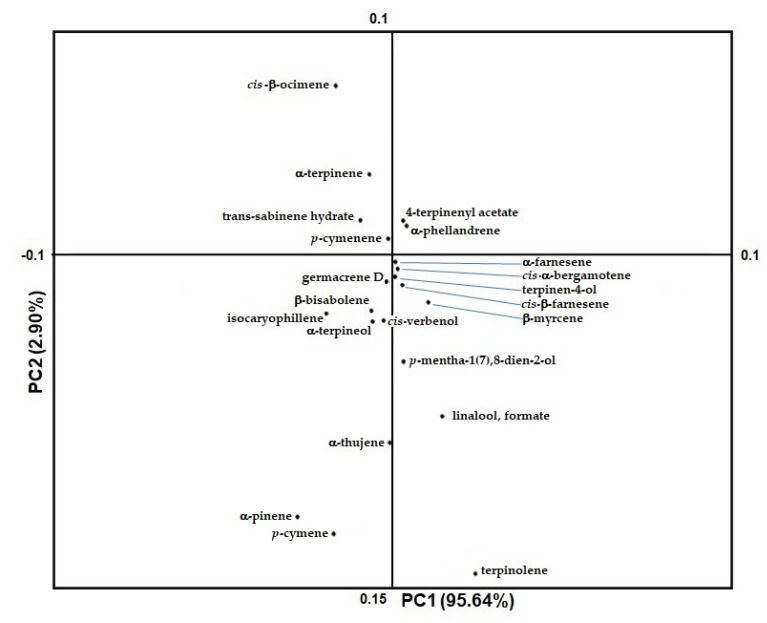
Detail of loading plot of volatile products extracted from citrus samples (juice and peel).

The loading plot (Figure 2b) is dominated by limonene in the second quadrant of the graph, and by γ-terpinene in the first quadrant of the graph. They are by far the first and second most abundant compounds, respectively, and are responsible for separation of juice samples. On the other hand, the most relevant metabolites responsible for the separation between juices and peels were found to be *cis*-β-ocimene, terpinolene, carveol, β-myrcene and α-citral. All other compounds (20 variables) are grouped around the center of the graph and most of them in the negative area of the Y axis (Figure 3). They give a small but significant contribution, due to their low concentration, in the separation of juice and peel samples.

### 2.2. Total Polyphenols and Flavonoids Content

Phenolic and flavonoidic content in the juices of the four citrus fruits is shown in Table 2. The levels of total polyphenols as determined by the Folin–Ciocalteu method varied from 125.2 to 219.5 mg/100 mL gallic acid equivalents, while the total flavonoids detected by the aluminum chloride method ranged from 1.9 to 5.1 mg/100mL quercetin equivalents. Oni Yuzu was found to be the significantly richest juice in both compound classes. The lowest amounts of polyphenols and flavonoids were found in Florence cedar and Bizzarria orange, respectively. Furthermore, a positive correlation between antiradical power and total phenolic or flavonoid content of the juices was also found. The highest degree of correlation was recorded between polyphenol or flavonoid amount and scavenging effect on the DPPH^•^ stable free radical, rather than on the ABTS^•+^ radical monocation (R^2^ = 0.635 and 0. 563 vs. 0.547 and 0.446, respectively).

### 2.3. Antiradical Activity

The effect of the different juices against ABTS^•+^ and DPPH^•^ radicals is summarized in Table 3. Despite the variability in the content of polyphenols and flavonoids among the four samples, three of them (Yuzu Jeune, Oni Yuzu and Bizzarria orange) exhibited similar values in terms of antioxidant activity (213.0 to 242.0 and 2.05 to 30.0 µM eq. Trolox/100 mL, respectively) with a good correlation between the two tests (Yuzu Jeune > Oni Yuzu > Bizzarria orange). Florence cedar appeared the least active toward both ABTS^•+^ and DPPH^•^. The related obtained data were two to three times lower than those of the other juices.

## 3. Discussion

The purpose of this study was to describe and compare the volatile chemical profile of the juices and peels from four uncommon citrus fruits (Yuzu Jeune, Oni Yuzu, Bizzarria orange, Florence cedar) grown in Switzerland. To this aim, the aromatic compounds both from the juices and peels were extracted and concentrated by solid-phase microextraction of the static headspace and subsequently separated and identified using GC/MS technique.

The volatile chemical composition of the juices and/or peels of some citrus fruits has been previously reported. However, it was determined in samples including that from *C. junos* obtained using solvent-based extraction processes [13,14,15]. Few works refer to the use of SPME technique to characterize volatile content in *Citrus* species. Among them, Cheong at al. [16] described a similar composition for the flower and peel of the white and pink Malaysian pomelo, with traces of compounds such as β-sinensal, α-sinensal and nootkatone found only in the white pomelo peel. Gonza’lez-Mas and collaborators [17] studied the volatile fraction of the fruit juice from Powell Navel orange, Clemenules mandarin, Fortune mandarin and Chandler pummelo, showing mainly quantitative differences. Barboni and co-authors [18] documented differences both qualitative and quantitative between juice and peel of the same sample in a study carried out on clementine, mandarin and their hybrids. Recently, these data on *C. reticulata* were confirmed by Figueira et al. [19], highlighting that the peels were richer in volatile components than juices. In our study, some quantitative differences were observed between the four species, especially with regard to the number of identified compounds (higher in Bizzarria orange and Florence cedar) and the abundance of the main compound (lower in Florence cedar).

In general, all the volatile fractions were dominated by monoterpene hydrocarbons, consistent with data of previous works [16,17,18,19]. In all cases, such prevalence was mainly due to limonene and γ-terpinene, in variable percentages, followed by β-pinene, *p*-cymene, terpinolene and other minor compounds. Some of the latter compounds were particular to a single citrus fruit, present in both analyzed parts or in only one. According to some authors, despite their low content, these compounds manage to actively contribute to the aroma [20].

On the other hand, terpenoids are believed to exert a protective or disease-preventing action [21,22,23,24,25,26,27], and additionally, phenolic compounds can provide a range of activities including the protection against oxidative damage [28,29]. In particular, polyphenols are the most well-known phytochemicals able to significantly decrease the adverse effects of free radicals [30]. The presence of these classes of compounds was verified in our samples and, therefore, the antioxidant capacity was investigated as an indicator of their in vitro potential as health promoters [31]. The total phenolic content appeared to be in line with or higher than that of other citrus juices [32,33,34]. Conversely, the flavonoid content was similar or lower [33,34]. As for antiradical ability, our juices proved to be inferior in scavenging action against the DPPH^•^ radical and showed comparable activity towards the ABTS^•+^ radical [33]. However, as it is known, the considered parameters can be influenced by various aspects such as the different starting material and the collection period, as well as different extraction and analysis methods, in addition to the unit of measurement [35,36,37].

Among the identified volatiles, some of them were previously tested for their antioxidant properties. For example, limonene showed a concentration-dependent reduction in free radical formation through various in vitro assays [38]. Tested together with other terpenoids, it has been shown to be among the most potent scavengers of the free radical DPPH as well as having one of the highest reducing powers [39]. Its antioxidant activity counteracts neuronal suffering induced by some oligomers, preventing the hyperactivity of KV3.4 in Alzheimer’s disease context [40]. In addition, *C. limon* (L.) Osbeck essential oil characterized by a composition rich in limonene presented values of antiradical activity comparable to that of the standard [41]. In a similar way, essential oils with γ-terpinene as one of the main volatile components exhibited appreciable antioxidant and antiradical activity based on several assays including DPPH and ABTS tests [42]. Reactions of the pure compound with ABTS^•+^ and DPPH^•^ implied that γ-terpinene was able to directly scavenge radicals as well as protect methyl linoleate, DNA and erythrocytes from oxidation induced by 2,20-azobis (2-amidinopropane hydrochloride) [43]. Previously, Foti and Ingold [44] proved an unusual mechanism of inhibition of lipid peroxidation by γ-terpinene via a very fast cross-reaction between HOO^•^ and LOO^•^ radicals. Other terpenoids such as *p*-cymene and β-pinene were detected as main constituents of some essential oils active against DPPH^•^ or showing different antioxidant abilities [42,45,46]. In addition, juices contain a variety of active substances, which could interact and affect the overall antiradical activity. The possible contribution by a multicomponent fraction including compounds present in smaller amounts rather than single antioxidants should not be overlooked.

## 4. Materials and Methods

### 4.1. General

Folin–Ciocalteau’s phenol reagent, 2,2-diphenyl-picryl hydrazyl (DPPH), 2,21-azino-*bis*(3-ethylbenzothiazoline-6-sulfonic acid) (ABTS), saturated sodium carbonate, aluminum chloride, potassium acetate, potassium persulfate, 6-hydroxy-2,5,7,8-tetramethychroman-2-carboxylic acid (Trolox), gallic acid and quercetin as well as ethanol and methanol were obtained from Sigma-Aldrich (Milan, Italy).

### 4.2. Citrus Materials (Peels and Juices)

All tested *Citrus* species were grown in the Nyon region, Switzerland. Fruits were harvested in October 2020 and stored in a fridge (about 5 °C) until analysis. Before use, the fruits were washed with deionized water and a hand extractor was used to obtain the juice with the intent of preventing any contamination by the peel components.

### 4.3. SPME of Peels and Juices

Thin slices of peels (~2 g) and juice (~2 mL) were individually placed into a 20 mL glass vial with PTFE-coated silicone septum. Samples were equilibrated for 30 min at 40 °C prior to analysis. For the sampling a SPME device from Supelco (Bellefonte, PA, USA) with 1 cm fiber coated with 50/30 μm DVB/CAR/PDMS (divinylbenzene/carboxen/polydimethylsiloxane) was used. After an initial conditioning phase of the fiber, at 270 °C for 20 min, it was exposed to the equilibrated samples headspace for 30 min 40 °C to capture the volatile compounds. Later, the SPME fiber was inserted in a GC injector maintained at 250 °C for the desorption of the compounds and then transferred to a GC column for subsequent separation.

### 4.4. GC-MS and GC-FID Chemical Analysis

The chemical analyses of the headspace from juices and peels were carried out on Clarus 500 model Perkin Elmer (Waltham, MA, USA) gas chromatograph coupled with a mass spectrometer and equipped with an FID (flame detector ionization). For the separation, a Varian Factor Four VF-1(Phenomenex, Torrance, CA, USA) capillary column was used. The temperature program was as follows: 40 °C for 2 min and then increasing to 220 °C at 6°/min and finally held for 10 min. Helium was used as carrier gas at a flow rate of 1 mL/min. The mass spectra were recorded at 70 eV (EI) and were scanned in the range 40–400 *m*/*z*. Ion source and the connection parts temperature was 220 °C.

For the identification of separated compounds, the matching of their mass spectra with those stored in the Wiley 2.2 and Nist 02 mass spectra libraries database was performed; furthermore, the Linear Retention Indices (LRIs), were calculated using a mixture of linear C8–C30 alkanes and compared with available retention data reported in the literature. All the identified compounds were all those detected in both the peels and the juices. The peak areas of the FID signal were used to calculate the relative percentages of the components without the use of an internal standard and any factor correction. All analyses were carried out in triplicate.

### 4.5. Determination of Total Polyphenols

Total polyphenols of the juices were spectrophotometrically quantified by the Folin–Ciocalteau assay [47]. Briefly, a suitable aliquot of the juice was combined with 50 μL of Folin–Ciocalteau reagent. After 3 min, 100 μL of a saturated sodium carbonate solution was added and the final volume was made up to 2.5 mL with distilled water. The solution was incubated in the dark for 1 h at room temperature, then its absorbance was read at 725 nm. Test was performed in triplicate and repeated three times. Results were expressed as mg gallic acid equivalents (GAE) per 100 mL of juice.

### 4.6. Determination of Total Flavonoids

The total flavonoid content was determined using aluminum chloride colorimetric method [48]. Hence, 500 μL of juice was placed in a test tube and 1.5 mL of methanol was added followed by 100 μL of AlCl_3_ (10% *w*/*v*) and 100 μL of potassium acetate (1 M). Then, 2.8 mL of distilled water was added to make the total volume up to 5 mL. The final solution was vortexed and incubated in the dark for 30 min at room temperature. Subsequently, the absorbance was read at 420 nm. Test was performed in triplicate and repeated three times. Results were expressed as mg quercetin equivalents (QE) per 100 mL of juice.

### 4.7. Determination of Antiradical Activity

#### 4.7.1. DPPH^•^ Radical-Scavenging Assay

The 2,2-diphenyl-picryl hydrazyl (DPPH^•^) radical-scavenging capacity was performed following Fracassetti et al. [49] with some modifications. In brief, the DPPH solution was diluted with methanol to obtain 1.00 ± 0.03 absorbance units at 515 nm. In a test tube, 2.45 mL of this solution was placed and 50 µl of juice was added. After 50 min of incubation in the dark at room temperature, the absorbance was read at 515 nm. Test was performed in triplicate and repeated three times. Results were expressed as µM Trolox mL^−1^ of juice.

#### 4.7.2. ABTS^•+^ Radical-Scavenging Assay

The 2,21-azino-*bis*(3-ethylbenzothiazoline-6-sulfonic acid) (ABTS^•+^) radical cation-scavenging capacity was determined as previously reported [50]. The ABTS^•+^ radical cation was produced by reacting 7 mM ABTS^•+^ with 2.45 mM potassium persulfate (final concentration) and maintaining the mixture in the dark at room temperature for at least 6 h before use. The ABTS^•+^ solution was diluted with ethanol to obtain 0.7 ± 0.02 absorbance units at 734 nm and equilibrated at 30 °C. Ten µL of each juice, ethanol (negative control) and standard solution of the synthetic antioxidant 6-hydroxy-2,5,7,8-tetramethychroman-2-carboxylic acid (Trolox, positive control) were mixed for 30 s with one mL of diluted ABTS^•+^ solution, respectively. Their absorbance was read at 734 nm, at room temperature, 50 s after the initial mixing. Test was performed in triplicate and repeated three times. Results were expressed as µM Trolox mL^−1^ of juice.

### 4.8. Statistical Analysis

All the results were expressed as means ± standard deviation (SD) and the ANOVA test (one-way analysis of variance test) followed by Tukey’s HSD test was used to analyze significant differences among means (*p* < 0.05). PCA was performed using the software R PACKAGE for Multivariate and Spatial Analysis (version 4.0) [51], on all the relative percentages of the identified compounds without scaling the values (matrix of covariances).

## 5. Conclusions

The chemical analyses carried out by SPME-GC/MS technique highlighted the presence of limonene and γ-terpinene as characterizing compounds of the aroma of these less common *Citrus* species. A detailed description of the chemical composition obtained through the identification of minority compounds, detected with lower percentages, allowed this research to highlight the differences in the volatile profile of the different investigated *Citrus* species. The results obtained showed that the SPME as a sampling method is appropriate and suitable to describe and compare the volatile chemical compositions of the juices and peels.

Regarding the antioxidant activity, our data, although preliminary, confirmed the generally reported free-radical-scavenging properties of citrus juices. All the identified active molecules work in synergy to exert their antiradical effect. Furthermore, together with other nutrients such as vitamin C, minerals or fibers, they are also responsible for the beneficial properties for human health thus representing an essential element in the food diet. Therefore, the unusual citrus fruits, increasingly popular among chefs, could soon have a wider market than the local one, as well as for their exotic and peculiar characteristics of shape and flavor, and as functional foods rich in natural health supplements.

## Figures and Tables

**Table 1:** Chemical composition (percentage mean value ± standard deviation) of vapor phase Yuzu Jeune, Oni Yuzu, Bizzarria orange and Florence cedar juice and peel.

N°	Component ^1^	LRI ^2^	LRI ^3^	Yuzu Jeune Juice (%)	Yuzu Jeune Peel (%)	Oni Yuzu Juice (%)	Oni Yuzu Peel (%)	Bizzarria Orange Juice (%)	Bizzarria Orange Peel (%)	Florence Cedar Juice (%)	Florence Cedar Peel (%)
1	α-thujene	918	923	0.69 ± 0.02^a^	0.68 ± 0.03^A^	0.20 ± 0.01^b^	-	0.09 ± 0.01^c^	0.35 ± 0.01^B^	-	0.80 ± 0.01^C^
2	α-pinene	940	943	-	-	-	0.80 ± 0.01^A^	0.75 ± 0.01^a^	1.00 ± 0.01^B^	0.88 ± 0.01^b^	1.81 ± 0.01^C^
3	camphene	942	946	-	-	-	-	-	tr	-	-
4	β-myrcene	978	983	0.78 ± 0.01	1.40 ± 0.02	-	-	-	-	-	-
3	β-pinene	982	986	1.13 ± 0.02^dc^	1.48 ± 0.02^D^	1.17 ± 0.01^c^	2.80 ± 0.01^B^	1.55 ± 0.02^a^	2.30 ± 0.02^C^	1.25 ± 0.02^b^	3.15 ± 0.01^A^
5	α-phellandrene	1000	^§^	0.96 ± 0.01^a^	0.44 ± 0.01^A^	0.19 ± 0.01^b^	tr	0.13 ± 0.01^cd^	0.10 ± 0.01^CB^	0.13 ± 0.01^d^	0.13 ± 0.01^B^
6	α-terpinene	1002	1008	-	-	-	0.15 ± 0.01^A^	-	0.14 ± 0.01^A^	0.54 ± 0.01	-
7	*p*-cymene	1020	1016	2.38 ± 0.01^a^	2.33 ± 0.01^BC^	0.59 ± 0.02^ca^	0.38 ± 0.01^D^	1.21 ± 0.01^b^	1.14 ± 0.01^C^	1.23 ± 0.01^db^	2.34 ± 0.01^A^
8	limonene	1025	1023	78.25 ± 0.02^b^	78.40 ± 0.02^B^	85.74 ± 0.02^a^	84.15 ± 0.02^A^	70.85 ± 0.01^c^	75.40 ± 0.02^C^	55.16 ± 0.02^d^	54.80 ± 0.02^D^
9	*cis*-β-ocimene	1029	1032	-	0.42 ± 0.01^A^	0.58 ± 0.02^b^	-	0.53 ± 0.01^c^	-	1.17 ± 0.02^a^	0.46 ± 0.02^A^
10	β-terpinene	1032	1036	-	-	-	-	0.11 ± 0.01	-	-	-
11	*trans*-sabinene hydrate	1048	1053	-	-	-	tr	0.29 ± 0.01^a^	-	0.34 ± 0.01^b^	0.28 ± 0.01
12	γ-terpinene	1051	1054	14.43 ± 0.01^c^	11.43 ± 0.01^C^	9.46 ± 0.01^d^	9.18 ± 0.01^D^	21.51 ± 0.02^b^	13.36 ± 0.02^B^	35.21 ± 0.01^a^	26.89 ± 0.02^A^
13	terpinolene	1067	1078	0.46 ± 0.02^c^	-	1.91 ± 0.01^a^	2.22 ± 0.02^A^	1.65 ± 0.01^b^	1.33 ± 0.02^C^	-	1.52 ± 0.01^B^
14	*p*-cymenene	1087	1083.4	-	-	-	-	-	-	0.11 ± 0.02	-
15	*cis*-verbenol	1125	1130	-	-	-	-	-	0.15 ± 0.01^B^	-	0.26 ± 0.02^A^
16	terpinen-4-ol	1158	1160	-	-	-	-	-	0.23 ± 0.01		-
17	*trans*-3-caren-2-ol	1165	1160	-	-	-	-	-	-	0.16 ± 0.01	-
18	α-terpineol	1181	1183	-	-	-	-	0.79 ± 0.01^a^	0.51 ± 0.02^A^	0.92 ± 0.01^b^	0.46 ± 0.02^B^
19	*p*-mentha-1(7),8-dien-2-ol	1189	1186	-	-	-	-	-	1.09 ± 0.02	-	tr
20	carveol	1198	1201	-	-	-	-	-	-	0.11 ± 0.01	1.98 ± 0.01
21	linalool, formate	1202	1206	-	2.81 ± 0.02	-	-	-	-	-	-
22	β-citronellol	1214	1212	-	-	-	-	-	tr	-	tr
23	perillal	1275	1277	-	-	-	-	-	tr	-	-
24	α-citral	1291	1287	-	-	-	-	-	1.66 ± 0.01^B^	0.21 ± 0.01	2.91 ± 0.02^A^
25	4-terpinenyl acetate	1300	1304	0.76 ± 0.00	-	-	-	-	-	-	-
26	δ-elemene	1340	1347	-	-	-	tr	-	-	-	-
27	isocaryophyllene	1375	1379	-	-	-	-	-	-	0.64 ± 0.01	0.78 ± 0.01
28	α-copaene	1381	1379	-	-	-	-	-	tr	-	-
29	*cis*-α-bergamotene	1402	1407	0.16 ± 0.01^b^	-			0.23 ± 0.01^a^	0.46 ± 0.02	-	-
30	*cis*-muurola-3,5-diene	1445	^§^	-	-	-	tr	-	-	tr	tr
31	*cis*-β-farnesene	1449	1451	-	0.53 ± 0.01	-	-	-	-	-	-
32	*cis*-muurola-4(14),5-diene	1460	^§^	-	-	-	-	-	-	tr	tr
33	germacrene D	1504	1500	-	-	-	-	-	tr	-	0.13 ± 0.01
34	β-bisabolene	1507	1501	-	-	-	-	0.15 ± 0.00^a^	0.22 ± 0.01^B^	0.13 ± 0.00^b^	0.37 ± 0.01^A^
35	α-farnesene	1509	1506	-	-	-	0.10 ± 0.03	-	-	-	tr
36	valencene	1512	1515	-	-	-	tr	-	-	-	-
37	δ-cadinene	1525	1530	-	-	-	tr	-	tr	-	-
38	SUM			100.0	99.92	99.84	99.78	99.84	99.44	98.19	99.07
	Monoterpene hydrocarbons			99.08	96.58	99.84	99.68	98.67	95.12	96.02	92.18
	Oxygenated monoterpenes			0.76	2.81	-	-	0.79	3.64	1.4	5.61
	Sesquiterpene hydrocarbons			0.16	0.53	-	0.10	0.38	0.68	0.77	1.28
	Others			-	-	-	-	-	-	-	-

^1^ The components are reported according to their elution order on apolar column; ^2^ Linear Retention indices measured on apolar column; ^3^ Linear Retention indices from literature (Nist Chemistry WebBook); ^§^ LRI not available; - Not detected; tr: traces: <0.1%. Lowercase letters represent Tukey’s Test comparison among juices and uppercase letters represent comparison among peels.

**Table 2:** Polyphenols and flavonoids of citrus juices.

*Citrus* Species	Total Polyphenols (mg GAE/100 mL Juice)	Total Flavonoids (mg QE/100 mL Juice)
Yuzu Jeune	146.0 ± 2.12	3.3 ± 0.06
Oni Yuzu	219.5 ± 0.71	5.1 ± 0.10
Bizzarria orange	165.0 ± 4.09	1.9 ± 0.10
Florence cedar	125.2 ± 3.07	2.2 ± 0.10

**Table 3:** Antiradical activity of citrus juices.

*Citrus* Species	ABTS (µM Trolox eq./100 mL Juice)	DPPH (µM Trolox eq./100 mL Juice)
Yuzu Jeune	242.0 ± 4.00	30.0 ± 0.00
Oni Yuzu	220.0 ± 2.02	29.0 ± 0.00
Bizzarria orange	213.0 ± 5.51	25.0 ± 0.00
Florence cedar	102.0 ± 110	10.0 ± 0.00

## Data Availability

All generated data are included in this article.

## Sample Availability

Samples of the compounds are available from the authors.
